# Research on Low-Frequency Noise Control of Automobiles Based on Acoustic Metamaterial

**DOI:** 10.3390/ma15093261

**Published:** 2022-05-01

**Authors:** Yi Liao, Haibo Huang, Guangbao Chang, Deyang Luo, Chuanlai Xu, Yudong Wu, Jiyou Tang

**Affiliations:** 1 SAIC GM WULING Automobile Co., Ltd., Liuzhou 545005, China; yi.liao1@sgmw.com.cn (Y.L.); guangbao.chang1@sgmw.com.cn (G.C.); deyang.luo@sgmw.com.cn (D.L.); 2 School of Mechanical Engineering, Southwest Jiaotong University, Chengdu 610031, China; huanghaibo214@my.swjtu.edu.cn (H.H.); jyt@my.swjtu.edu.cn (J.T.); 3 Sichuan Jiuzhou Electric Group Co., Ltd., No. 6 Jiuhua Road, Mianyang 621000, China; 4 Sichuan Avionics System Product Lightweight Design and Manufacturing Engineering Laboratory, Mianyang 621000, China; 5 National Laboratory of Rail Transit (in Preparation), Chengdu 610031, China

**Keywords:** acoustic metamaterial, local resonance, low-frequency, roar noise control, steady-state noise

## Abstract

With the transformation of the trend of vehicle electrification, the overall noise level in the vehicle is gradually reduced. The problem of low-frequency noise in the vehicle, which was previously ignored, is becoming more and more prominent. To solve the vehicle low-frequency noise problem, a combination of real-vehicle tests and simulation analysis is carried out. During the test, the driver and passengers feel that there is a relatively obvious low-frequency roar noise in the car, which results from the structural radiation noise of the trunk door vibration. Therefore, to solve this problem, we design an acoustic metamaterial with lightweight and miniaturized features based on the local resonance principle of phononic crystals. Firstly, the selection of the resonant unit configuration and the design of the band gap are implemented. Then, the layout planning of the whole vehicle, the layout of the resonance unit and the design of the base frame are implemented. The actual vehicle test results show that: after attaching the designed acoustic metamaterial, the low-frequency noise sound pressure levels in the front and rear of the vehicle were reduced by 2.0 dB (A) and 2.3 dB (A), respectively, meanwhile, the interior noise sound quality was improved. The sound pressure level at the driver’s right ear in the car has an abnormal peak of around 35Hz. In addition, the driver and passengers feel that there is a relatively obvious low-frequency roar noise in the car, and through low-pass filtering of the collected signals, it is confirmed that the peak frequency is the main cause of the low-frequency roar in the car. The low-frequency steady-state noise of the car is generally considered to be the low-frequency vibration of the body panel and the radiation occurs. Through the finite element simulation analysis (Grid Participation Analysis) of the abnormal peak frequency, the results show that the low-frequency roar is caused by the low-frequency vibration of the tailgate sheet metal, and the problem peak frequency is not coupled with the acoustic cavity mode. Facing the problem of the low-frequency roar radiated into the car by the vibration of the tailgate sheet metal parts, based on the local resonance band gap theory, we developed a design to suppress the 35 Hz vibration of the tailgate sheet metal parts and meet the characteristics of lightweight and miniaturization. By attaching the acoustic metamaterial to the tailgate and performing CAE simulation of the whole vehicle, it is determined that the structure can indeed reduce the 35 Hz noise in the car and the peak value of the tailgate sheet metal vibration.

## 1. Introduction

**Figure 1:**
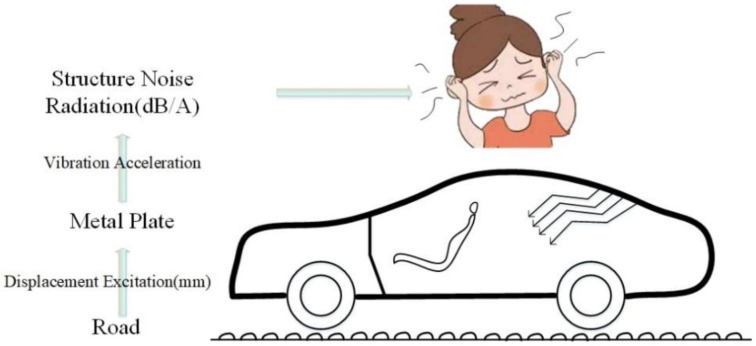
Noise radiation path of rough road structure (tailgate).

In the field of automotive NVH (Noise, Vibration, Harshness), high-frequency noise and vibration control can achieve ideal results through acoustic packaging or attaching vibration damping, but controlling the radiation caused by the vibration of automotive sheet metal parts low-frequency noise has always been a major and difficult problem in the field of automotive noise and vibration control [1,2,3]. For automobiles, the low-frequency noise and vibration problems radiated into the car caused by the vibration of sheet metal parts will not only seriously affect the ride comfort of the driver and passengers, but also have a great impact on the driving safety and service life of the car [4]. In order to obtain a more comfortable and safe driving and riding environment, and to improve the market competitiveness of vehicles, it is extremely important to control the low-frequency noise radiated into the vehicle by the vibration of the automobile sheet metal parts [5]. The generation path of low-frequency noise radiated by automobile (tailgate) sheet metal parts is shown in Figure 1. The car is driving on rough roads, and the displacement excitation of the road is transmitted to the body sheet metal parts through the tire–suspension system [6]. Excitation generates low-frequency vibration and radiates low-frequency structural noise into the car, causing serious discomfort to passengers and drivers [7,8].

At present, there are three main ways to reduce the vibration of sheet metal parts in response to the problem of low-frequency noise radiated by the vibration of automobile sheet metal parts at home and abroad [9,10]. First, using spring-damping vibration-reducing materials or rubber vibration-reducing materials. With the development of current industrial technology, it is found that the use of spring-damping vibration-reducing materials or rubber damping vibration-reducing materials has a more obvious effect on high-frequency vibration, but it is difficult to achieve an ideal suppression effect of the middle and low-frequency noise [11]. Second, applying reinforcing ribs to the sheet metal parts to increase the natural frequency of the low-frequency band of the sheet metal structure by increasing the rigidity, but the structure of the automobile sheet metal parts is greatly changed which will cause the production cost of the model to increase sharply, and seriously affect the economic benefits of the enterprise [12]. Third, installing the dynamic vibration absorber, but its cost is higher and the mass is higher [13]. In view of the above problems, the local resonance type vibration-damping acoustic metamaterial with negative dynamic equivalent parameter characteristics provides a new idea for noise control of low-frequency vibration radiation of automobile sheet metal parts [14,15,16,17,18,19].

In recent years, studies on the locally resonant phonon crystal formed by the periodic arrangement of elastomers and mass scatterers have been studied with great vigor [20]. Since the band gap produced by it is not limited by the size of the unit cell, it has a broad application market for low-frequency vibration and noise reduction in automobiles. In 2000, when Liu Zhengyou et al. studied the three-dimensional phononic crystal formed by the cubic lattice structure of lead balls coated with viscoelastic silicone rubber soft material and embedded in epoxy resin, they found that the wavelength of the phononic crystal forbidden band is much larger [21]. Due to the size of the lattice, it breaks through the limitation of the Bragg scattering mechanism and can control elastic waves two orders of magnitude lower than the resonant unit. Moreover, the scatterers are not strictly periodic or even randomly distributed, the composite structure also has a band gap effect [22]. This puts forward the local resonance mechanism of the elastic wave band gap, which provides a new theoretical basis for the follow-up automotive low-frequency noise and vibration control field [23]. In 2001, BAZ introduced active control to study the transmission characteristics of elastic waves in periodic beams with two materials alternately arranged, gave the corresponding simplified theoretical analysis model of the spring–mass system, explained the active adjustment method of passband and forbidden band, and proposed the directional design method of local resonance type acoustic metamaterial [24]. In 2008, Hong Kong University of Science and Technology Yang Zhiyu and others proposed a thin-film acoustic metamaterial based on the local resonance theory [25,26,27]. The lightweight structure with a thickness as low as millimeters successfully achieved outstanding sound insulation capabilities in the low-frequency range of 500~800 Hz, which provided a new solution for low-frequency noise reduction. The elastic film needs external tension to propagate the vibration [28]. As the stiffness of the elastic film is very low and the performance is unstable, a small change in tension will cause a frequency shift of tens or even hundreds of hertz. Therefore, no matter from a theoretical or technical point of view, effective control of film tension is very necessary. In 2015, Ma Fuyin and others at Xi’an Jiaotong University proposed a square lattice thin-film acoustic sound insulation metamaterial that requires almost no tension by increasing the thickness of the film [29]. In 2019, Niu Jiamin and others from Xi’an Jiaotong University proposed that under the asymmetric mode, the overall sound absorption performance of the structure has been greatly improved, and the sound absorption bandwidth has been expanded from the original narrow frequency to a wide frequency [30].

In summary, although local resonance acoustic metamaterials seem to be relatively new products now, there are a lot of data on the research of its mechanism, and there are also certain physical samples to verify its vibration suppression effect. However, the current acoustic metamaterials developed by technical institutions have poor stability and the vibration suppression frequency is difficult to reduce to less than 100 Hz. The acoustic metamaterial research is almost limited to simulation design and theoretical research.

Focusing on the problem of the low-frequency roar radiated into the car by the vibration of the tailgate sheet metal of a certain vehicle model, based on the theory of local resonance bandgap, a design is designed to suppress the 35 Hz vibration of the tailgate sheet metal with stable and small equivalent stiffness. Through the vehicle finite element simulation, it is verified that the structure can indeed reduce the 35 Hz noise peak in the vehicle and reduce the vibration of the tailgate sheet metal parts, thereby providing a new idea and new solution for the field of vehicle low-frequency noise and vibration control.

## 2. Basic Theory of Local Resonance Acoustic Metamaterial

### 2.1. Elastic Wave Equation in Acoustic Metamaterial

An acoustic metamaterial is a kind of man-made periodic composite structure material, which is developed from crystals in solid mechanics [31,32]. Therefore, the theory of the energy band of crystals in solid mechanics is also applicable to periodic composite structures. The content of acoustic metamaterial exploration is the propagation of elastic waves in the medium, and the band gap is due to the special effects of elastic waves when they are transmitted in the structure [33]. According to the knowledge of elastic dynamics theory, under the condition of ensuring an ideal elastic medium, small displacement and no initial stress, consider any particle in the homogeneous acoustic metamaterial medium, and use the displacement method used in solving dynamic problems to accurately establish three types of equations describing the relationship between the mass point force, displacement, stress and strain in the acoustic metamaterial are as follows:

Differential Equation of Motion:

(1) $$\frac{\partial \sigma_{x}}{\partial x}+\frac{\partial \tau_{yx}}{\partial y}+\frac{\partial \tau_{zx}}{\partial z}+\rho x=\rho \frac{\partial^{2}u}{\alpha t^{2}}$$

(2) $$\frac{\partial \tau_{xy}}{\partial x}+\frac{\partial \sigma_{y}}{\partial y}+\frac{\partial \tau_{zy}}{\partial z}+\rho y=\rho \frac{\partial^{2}v}{\alpha t^{2}}$$

(3) $$\frac{\partial \tau_{xz}}{\partial x}+\frac{\partial \tau_{yz}}{\partial y}+\frac{\partial \sigma_{z}}{\partial z}+\rho z=\rho \frac{\partial^{2}w}{\alpha t^{2}}$$

Geometric Equation:

(4) $$\varepsilon_{x}=\frac{\partial u}{\partial x}\;\;\gamma_{yz}=\frac{\partial w}{\partial y}+\frac{\partial v}{\partial z}$$

(5) $$\varepsilon_{y}=\frac{\partial v}{\partial y}\;\;\gamma_{zx}=\frac{\partial u}{\partial z}+\frac{\partial w}{\partial x}$$

(6) $$\varepsilon_{z}=\frac{\partial w}{\partial x}\;\;\gamma_{xy}=\frac{\partial v}{\partial x}+\frac{\partial u}{\partial y}$$

Physical Equation:

(7) $$\sigma_{x}=\lambda \theta_{t}+2\mu \varepsilon_{x}\;\tau_{yz}=\mu \gamma_{y}$$

(8) $$\sigma_{y}=\lambda \theta_{t}+2\mu \varepsilon_{y}\;\tau_{zx}=\mu \gamma_{z}$$

(9) $$\sigma_{z}=\lambda \theta_{t}+2\mu \varepsilon_{z}\;\tau_{xy}=\mu \gamma_{x}$$

The above equation involves the six components of the stress tensor ($\sigma_{x}$, $\sigma_{y}$, $\sigma_{z}$, $\tau_{yz}$, $\tau_{zx}$, $\tau_{xy}$), six components of strain tensor ($\gamma_{xy},\gamma_{xz},\gamma_{yz},\varepsilon_{x},\;\varepsilon_{y},\varepsilon_{z}$) and three displacement components (*u*, *v*, *w*), a total of 15 unknowns of spatial coordinates *x*, *y*, *z* and time t. $\sigma_{x}$ is the normal stress, $\tau_{xy}$ is the shear stress, ρ is mass point density, $\gamma_{x}$ is shear strain, $\varepsilon_{x}$ is the normal strain, λ,μ are Lame coefficients, $\theta_{t}=\varepsilon_{x}+\varepsilon_{y}+\varepsilon_{z}$.

Not every equation contains the unknowns in the above equation, by using a displacement method often used to solve the equation of elastic dynamics, considering some of these unknown function hypotheses into the mathematic expression of the “basic unknown functions”, it can be brought into the rest of the equation, and can obtain the expressed in the displacement of stress components, the final three displacements as unknown functions derived the elastic dynamics equation:

(10) $$\left(\lambda +2\mu \right)\nabla \left(\nabla *\vec{\mu }\right)-\mu \nabla *\nabla \vec{u}+\rho w^{2}\vec{u}=0$$

In the equation, $\vec{u}$ is the displacement vector; λ, μ are the Lame coefficient; ρ is the density of the medium; ∇ is the Laplace operator; the relation between them and the wave velocity is expressed as: Longitudinal wave velocity: $C_{l}=\sqrt{\left(\lambda +2\mu \right)/\rho }$, Shear wave velocity: $C_{t}=\sqrt{\mu /\rho }$.

### 2.2. Acoustic Metamaterial Band Gap Mechanism

**Figure 2:**
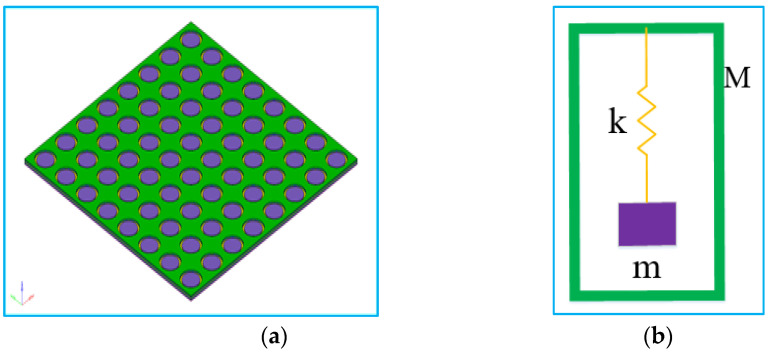
Typical local resonant acoustic metamaterial crystal cell structure and simplified model. (**a**) Typical acoustic metamaterial model, (**b**) Simplified acoustic metamaterial model.

When the vibrational elastic wave propagates in the local resonant phonon crystal, it will be affected by the periodic elastic scatterers, and the elastic wave within a certain intrinsic frequency range cannot continue to propagate through the elastic scatterers, which is called the forbidden band of the acoustic metamaterial. Other eigenfrequency ranges that can pass through these elastic scatterers without obstruction are called passbands. As shown in Figure 2a, it is a typical phonon crystal structure with local resonance. The structure is composed of mass scatterers, an elastic coating layer and matrix. The structure can be simplified to a typical spring–mass system, as shown in Figure 2b. The mass scatterers are composed of a dense metal material, which provides the mass component (expressed as m) for the cell structure. The elastic coating layer is composed of a thin film with certain elasticity, which provides the elastic component of the crystal cell structure (expressed as k). The matrix is rigid material (expressed as M), which is the carrier of vibration elastic wave transmission [34,35,36,37].

Suppose the displacement of matrix *M* and mass component m is *X* and *x*, the matrix excitation is *F*, and the reaction force of the mass component is $F_{1}$. By Newton’s second law and Hooke’s law:

(11) $$F-F_{1}={\left(jw\right)}^{2}MX$$

(12) $$F_{1}={\left(jw\right)}^{2}mx$$

(13) $$k\left(X-x\right)=F_{1}$$

Consider the base body and spring–mass system as a whole:

(14) $$m_{e}=M+\frac{mw_{0}{}^{2}}{w_{0}{}^{2}-w^{2}}$$

(15) $$H\left(w\right)=\frac{X}{F}=-\frac{1}{m_{e}w^{2}}$$

In the equation: $m_{e}$ is the system equivalent mass, H(w) is the system displacement frequency response function, ω is the natural circular frequency, w0=km is the natural frequency of the internal spring oscillator.

**Figure 3:**
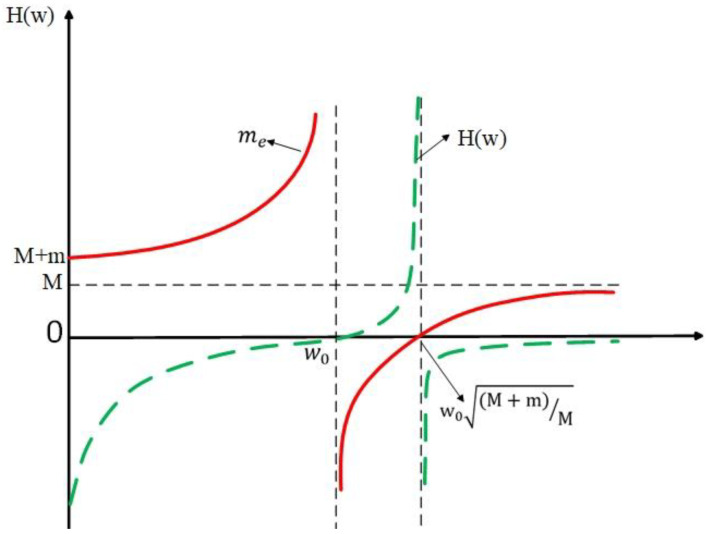
Relationship between external excitation frequency and displacement frequency response function.

It can be seen from the above formula that: with the change of external excitation frequency, the system shows different characteristics due to its different equivalent mass, as shown in Figure 3.

According to Newton’s second law and Hooke’s law theory and the external excitation frequency and displacement frequency response function, the relationship between the dynamic equivalent quality of the system and the excitation frequency can be inferred, as shown in Table 1.

In conclusion, the band gap is caused by the resonance of the internal mass component. The band gap range is determined by the natural frequency of the whole structure and internal mass component. The band gap effect occurs when the system is in the negative equivalent mass phase, the excitation frequency is in the range of [w0,w0(M+m)M]. Therefore, the frequency range generated by bandgap can be adjusted directionally by changing the mass or spring of the equivalent simplified model of the cell unit. Based on the above theory, according to the vibration frequency of auto sheet metal parts (equivalent to excitation frequency), an acoustic metamaterial can be designed to suppress the specific frequency.

## 3. Analysis of Low-Frequency Roaring in Car

**Figure 4:**
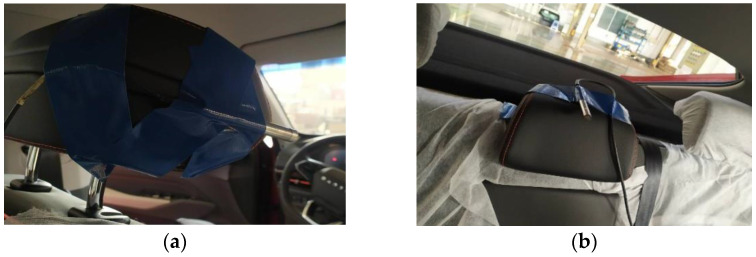
Position of sound pressure sensors. (**a**) driver’s seat, (**b**) passenger’s seat.

**Figure 5:**
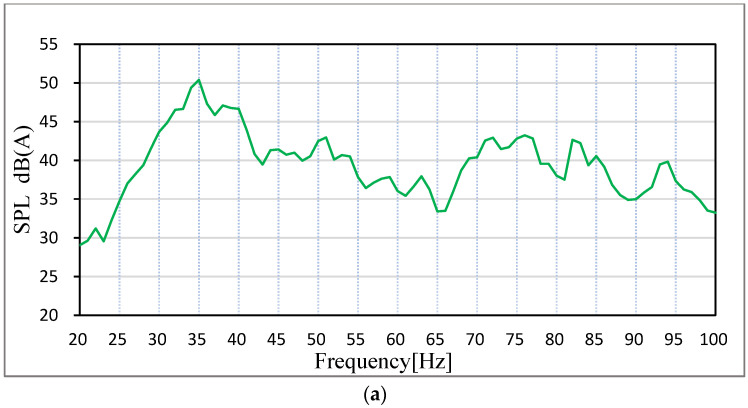

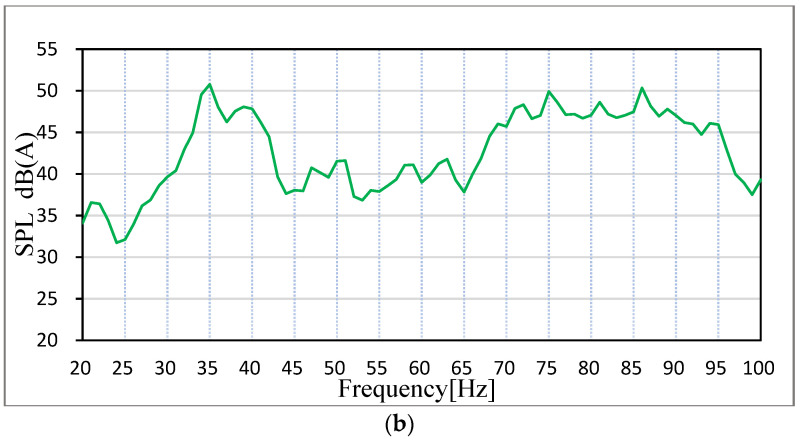
Spectrum diagram of noise in the car. (**a**) Driver’s seat, (**b**) Passenger’s seat.

Aiming at the relatively obvious low-frequency roar (20–100 Hz) generated by a certain hatch car when it is running at a constant speed of 30 km/h on a rough road surface, the LMS equipment is used to collect the sound pressure level at the driver’s right ear and at the middle back passenger seat. Sound pressure sensors are arranged according to relevant national standards GB/T18697-2002, and the location of measuring points is shown in Figure 4. During the signal acquisition process, the adjustable seat is back as far as possible to put it in a vertical position. The test is empty, with only the driver and measurement of personnel in the car. Before the test we ensure that the car is in a closed state, the four-wheel positioning is normal and in the test environment, the influence of background noise is less than the allowable error. When the vehicle speed is adjusted to a constant speed of 30 km/h, LMS onboard equipment is used to collect the sound pressure signal inside the vehicle. The noise test results are shown in Figure 5.

The test results show that the car has an obvious peak value at 35 Hz in the frequency range of 20–100 Hz. The peak value at 35 Hz in the right ear of the driver is 50.39 dB (A), and the peak value at 35 Hz in the middle of the passenger rear seat is 50.78 dB (A), as shown in Figure 4 (ordinate of Figure 4a is dB (A) and ordinate of Figure 4b is Pa). In the LMS test software, the collected signals were filtered to filter the peak value at 35 Hz, and the filtered noise signals were listened to by earphones, and it was found that the low-frequency roar had disappeared. Therefore, it can be determined that the low-frequency roar of this vehicle is caused by the peak noise at 35 Hz. As the peak frequency is very low, the continuous steady-state noise of low frequency is generally considered structural noise [38,39].

### 3.1. Analysis of Interior Noise Problem

**Figure 6:**
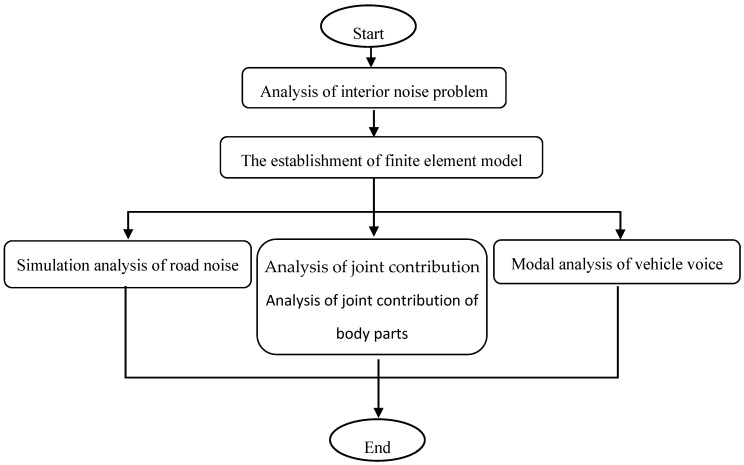
CAE diagnostic analysis flow of low-frequency roaring in the car.

Generally speaking, there are two ways to generate structural noise: the sound cavity mode of the whole vehicle and the coupled resonance of the body parts amplify the noise to form a low-frequency roar; the low-frequency vibration of body sheet metal radiates to the car, forming a low-frequency roar. In view of the above two approaches, this paper solves the acoustic cavity mode of the vehicle and analyzes the joint contribution of the panel parts based on the peak noise frequency of 35 Hz in the simulation analysis of road noise. The analysis process is shown in Figure 6.

Through CAE simulation analysis, the sound mode of the vehicle has no resonance with the body parts at 35 Hz. Therefore, the peak noise frequency of 35 Hz is mainly caused by the vibration radiation of the automobile panel.

#### 3.1.1. The Establishment of Finite Element Model

In order to carry out acoustic cavity modal analysis, road noise simulation and body plate joint contribution analysis of the whole vehicle, it is necessary to establish the sound cavity finite element model and acoustic–solid coupled finite element model.

**Figure 7:**
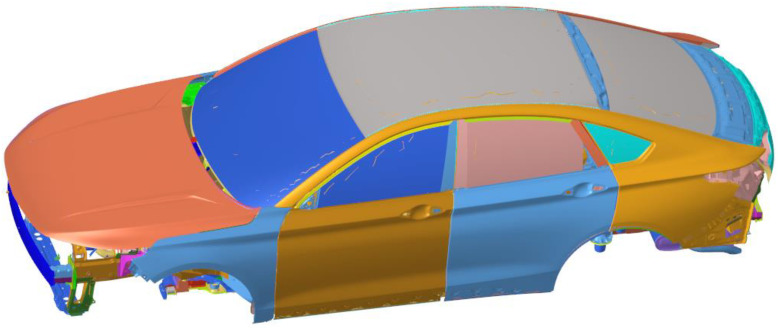
TB finite element model.

The TB (Trim Body) body is further constructed on the basis of the finite element model of body-in-white that has been constructed and modified through tests, which includes all parts except the engine and chassis system, many of which are replaced by centralized unit mass and one-dimensional unit. Determine the modeling standards and guidelines between the connections of various components: (1) Modeling of spot welding structural adhesives. Spot welding structural adhesives are mainly used in key parts of the car body, which can greatly improve the stiffness of the car body when used in conjunction with welding spots. Modeling of RBE3-hexa-RBE3 for spot welding of structural adhesive. (2) Modeling of shock-absorbing expansion adhesive. Shock-absorbing expansion adhesive mainly connects the outer plate of the top cover with the beam of the top cover, the outer plate of the open and closed parts with the support plate or the inner plate, and mainly plays a shock-absorbing role. (3) Modeling of reinforcement plate and damping plate. A reinforcement glue plate is generally used for side circumference and rear door plates with a thickness of 1.5 mm. It mainly plays the role of stiffness reinforcement and has a certain damping dissipation function. (4) Seal strip modeling. The seal strip is simulated using BUSH (used to define the nominal property values for a generalized spring and damper structural element) or ELAST (used to define the stiffness and stress coefficient of a scalar elastic element) in three translational directions. (5) Limit buffer block modeling. A limit buffer block is mainly used in the engine room cover and back door, to play a shock absorption and support role. The BUSH element is used to simulate and only three translational stiffnesses are set. (6) Connection between car door lock and lock. The BUSH unit can be used to simulate the model car door lock and lock. (7) Hinge modeling for open and closed parts. The hinge needs solid grid modeling, the grid size can be set to 3–5 mm, and the solid grid requires at least three layers of body elements in the direction of thickness. If the hinge is modeled using a solid grid or a shell element, the stiffness of the hinge will be overestimated. Doors generally contain two hinges connected by RBE2 and CBEAM, and the RBE2 center of the two hinges should be set to be collinear to ensure doors can rotate freely around the hinge. The hinge shaft is simulated using three CBEAM units and releases rotational degrees of freedom at both ends. The TB finite element model established is shown in Figure 7.

**Figure 8:**
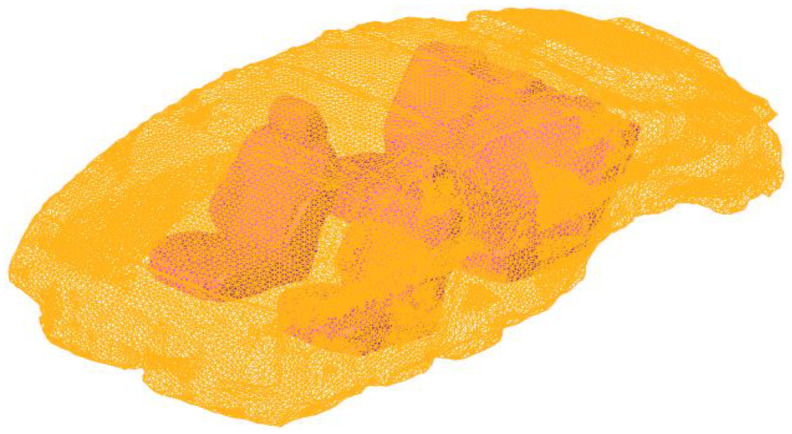
Acoustic cavity finite element model.

In Hypermesh 2017.01 software, Altair Engineering Inc. (Troy, MI, USA), we extract the inner surface of the body’s internal contact with air, to form a completely closed acoustic cavity model, which defines the spoke boundary as a fully rigid wall. When dividing the finite element mesh, it should be noted that the size of the acoustic element and the structural element should be consistent, and the acoustic element should have at least six elements in each wavelength range. As we are focusing on frequency within 100 Hz, the minimum wavelength *λ* = *c*/*f* = 344/100 = 3.44 m, and the maximum length of the sound cavity grid element is 0.52 m. In this paper, a hexahedral element with a side length of 50 mm is used to automatically divide the cavity mesh. The generated results of the finite element model of the body cavity are shown in Figure 8.

**Figure 9:**
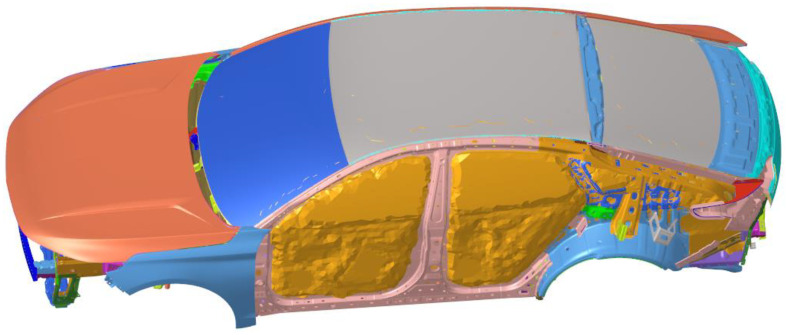
Sound–solid coupling finite element model of the body system.

The body structure and the sound cavity system are coupled through the vibration of the boundary nodes. In Hypermesh2017.01 software, the coupling between the structure and the sound cavity is realized by using the ACMODF card to search the structure nodes automatically through the nodes on the sound cavity boundary. The acoustic–solid coupling model of the body system is established as shown in Figure 9 (the left door and part of the side circumference are hidden to display the acoustic cavity model).

#### 3.1.2. Simulation Results of Road Noise

**Figure 10:**
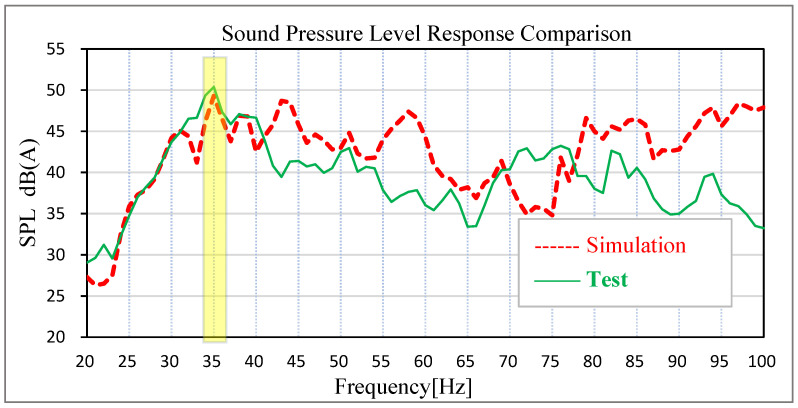
Comparison of test results and simulation results of driver’s right ear noise.

**Figure 11:**
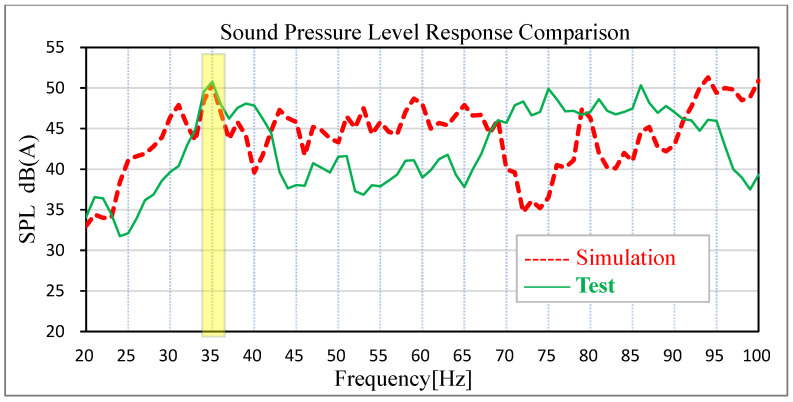
Comparison of test results and simulation results of passenger’s right ear noise.

Based on the acoustic–solid coupled finite element model, the time-domain signals of acceleration measured by engineers on the actual road surface at the steering knuckle of the front and rear suspension are converted into PSD frequency-domain signal files to stimulate the four wheel cores of the vehicle, and the solution response points are the sound pressure level at the driver’s right ear and the middle sound pressure level of the rear passenger’s seat. The solution frequency range is set as 20–100 Hz. The comparison between the test data and the simulation data of road noise is shown in Figure 10 and Figure 11.

The results show that the sound pressure level of the driver’s right ear and the middle of the passenger’s rear seat has an obvious peak value at 35 Hz in the low-frequency band, which is corresponding to the peak frequency of the test results of the real car and the overall trend is basically corresponding. The sound pressure level in the right ear of the real car test driver is 50.39 dB (A), and the simulation data is 49.30 dB (A). The test sound pressure level in the middle of the rear passenger row is 50.79 dB (A), and the simulation data is 50.50 dB (A). In the simulation and test, the difference in the sound pressure level between the driver’s right ear and the middle of the rear passenger’s row is relatively small at the peak point, indicating that the previous modified finite element model has high accuracy. In view of this kind of steady-state structural noise, the main contribution of the peak frequency can be effectively determined by analyzing the joint contribution of the body panel.

#### 3.1.3. Analysis of Joint Contribution of Body Parts

The control path of vehicle interior noise is generally: excitation source–transfer path–response point. For mass-produced models, it is very difficult to control the excitation source (chassis–suspension system). Modifying the chassis–suspension system will have a great impact on the smoothness, operation stability and safety performance of the whole vehicle, and the modification cost is huge. Therefore, for the noise problem of body vibration radiation, general enterprises tend to consider it from the perspective of transmission path (body sheet metal). The car body and the car sound cavity can be regarded as a closed cavity. The closed cavity sheet metal S low-frequency vibration radiates into the cavity to form the car’s low-frequency roar. Take a point inside the space O as the origin of coordinates, and the rest of the points in the space use it as a reference point. Assume that point *J* is a point on the closed cavity sheet metal part *S*, and its radiation sound pressure on a point *i* in the car is:

(16) $$P_{ij}\left(r_{0},w\right)=\frac{P_{j}\left(r,w\right)}{Q_{i}}v_{i}\left(r,w\right)\sigma S_{j}$$

where $P_{ij}\left(r_{0},w\right)$ is the radiation sound pressure from point *J* to point *I*, $Q_{i}$ is the volume sound source at point *i*, $P_{j}\left(r,w\right)$ is the sound pressure at point *j*, $v_{i}\left(r,w\right)$ is the speed that the sound source *j* transmits to point *i*, $r_{0}$ is the distance from point *i* to point *o*, *r* is the distance from point *j* to point *o*, *w* is the frequency. Many points on the body closed cavity sheet metal parts *S* radiation noise to point *i*, and the proportion of point *j* is:

(17) $$t_{j}\left(w\right)=\frac{P_{ij}\left(r_{0},w\right)}{P\left(r_{0},w\right)}$$

**Figure 12:**
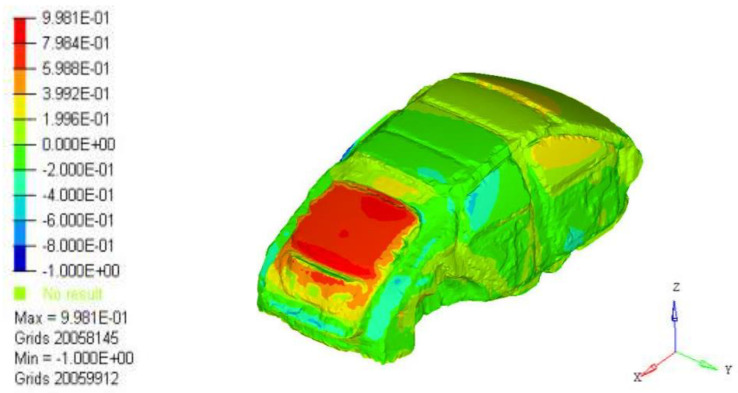
A 35 Hz cloud graph of plate joint contribution.

$P\left(r_{0},w\right)$ is the total sound pressure at point *i*, and $t_{j}\left(w\right)$ is the contribution of point *j* on the body closed cavity sheet metal part *S* to the interior noise. When all the point contributions are plotted together, the contribution of each area to the interior acoustic radiation can be determined. Through the analysis of the contribution source of the nodes, the main contribution node area can be found, so as to determine the source of the low-frequency roar contribution. Analysis results of 35 Hz body panel joint contribution were extracted, as shown in Figure 12. The results show that the rear door has the largest contribution to 35 Hz noise.

#### 3.1.4. Simulation Analysis of Vehicle Acoustic Cavity Mode

In the process of driving a car, the sheet metal parts of the car body and other sealing components form a closed cavity, and the acoustic cavity mode of the car reflects the inherent properties of the interior air fluid. When the structure resonates with the acoustic cavity mode, the interior noise will be amplified to form the low-frequency roaring sound (20 Hz–100 Hz). By comparing the result of free mode analysis of the sound cavity with the noise in the car, it can be judged whether the 35 Hz noise in the car is caused by the resonance between the sound cavity and the body panel.

**Figure 13:**
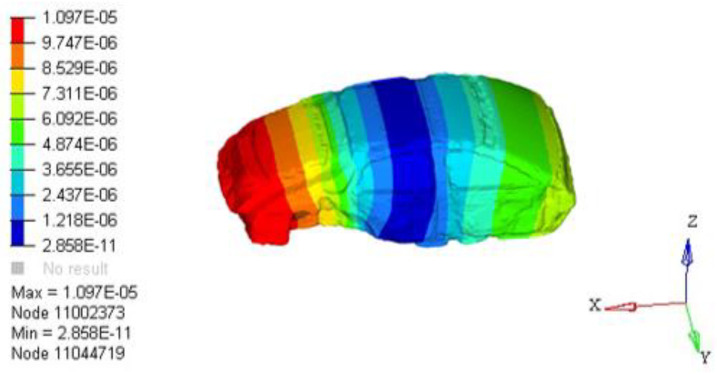
Model vibration shape of the first-order acoustic cavity.

**Figure 14:**
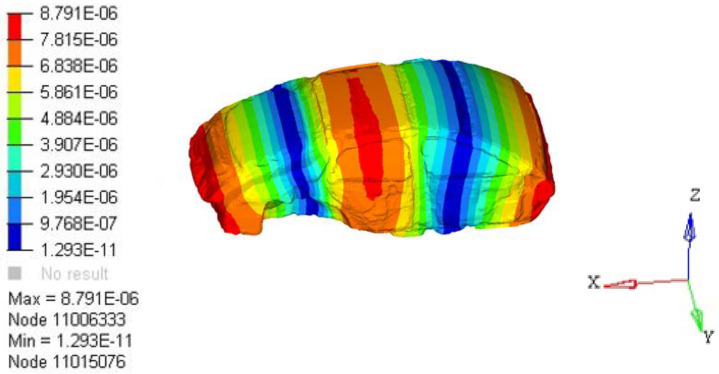
Model vibration shape of the second-order acoustic cavity.

The modal solution card was set up in the cavity finite element model established earlier, and the modal solution file was imported into Nastran14.0 solver for solving. Through calculation, the modal frequency and mode shape of the acoustic cavity of the structure are obtained in Table 2, Figure 13 and Figure 14:

### 3.2. Acoustic Metamaterial Design and Simulation Analysis

#### 3.2.1. Establishment of Mathematical Model of Acoustic Metamaterial

**Figure 15:**
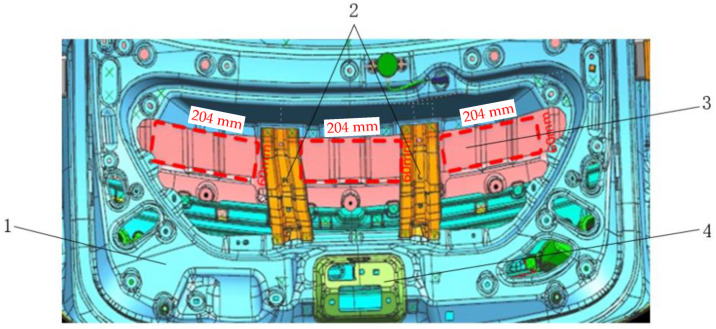
Installation position of acoustic metamaterial (red dotted box).

**Figure 16:**
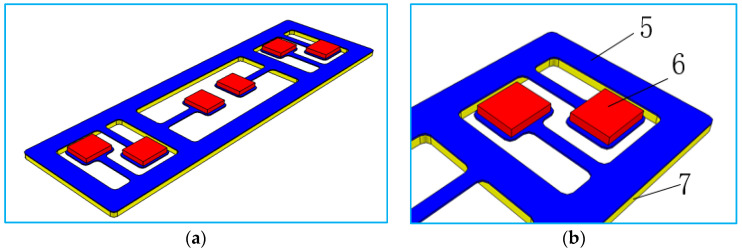
Mathematical model of acoustic metamaterial. (**a**) The designed local resonance acoustic metamaterial, (**b**) the local resonance unit.

Through simulation, the front section has determined that the peak noise of 35 Hz in the car is mainly caused by low-frequency vibration radiation of the tail door. Therefore, according to the corresponding size structure of the plate of the tail door, an acoustic metamaterial with specific frequency suppression can be designed. According to the mathematical model of the tailgate, the installation position of the acoustic metamaterial is determined corresponding to the position of the red box shown in Figure 15 (the rectangular shape of the acoustic metamaterial is 60 × 204 × 3 mm, 1 is the inner panel of the tail door, 2 is the reinforced floor of the tail door, 3 is the outer panel of the tail door, 4 is the car tail door lock, and the red dotted box is the attachment position of the acoustic metamaterial of the tail door). The acoustic metamaterial is composed of three parts: Acoustic metamaterial matrix plate 5, adjustable mass block 6 and acoustic metamaterial damping layer 7, as is shown in Figure 16. The designed acoustic metamaterial is directly attached to the sheet metal parts of the tailgate through the magnetic damping layer, eliminating the need for a complicated installation process. The adjustable mass block and the cantilever beam in the acoustic metamaterial layer constitute a typical acoustic metamaterial spring–mass system, which directionally regulates the band gap frequency of the acoustic metamaterial by adjusting the weight of the mass block.

#### 3.2.2. Frequency Design of Acoustic Metamaterial Resonance Element

**Figure 17:**
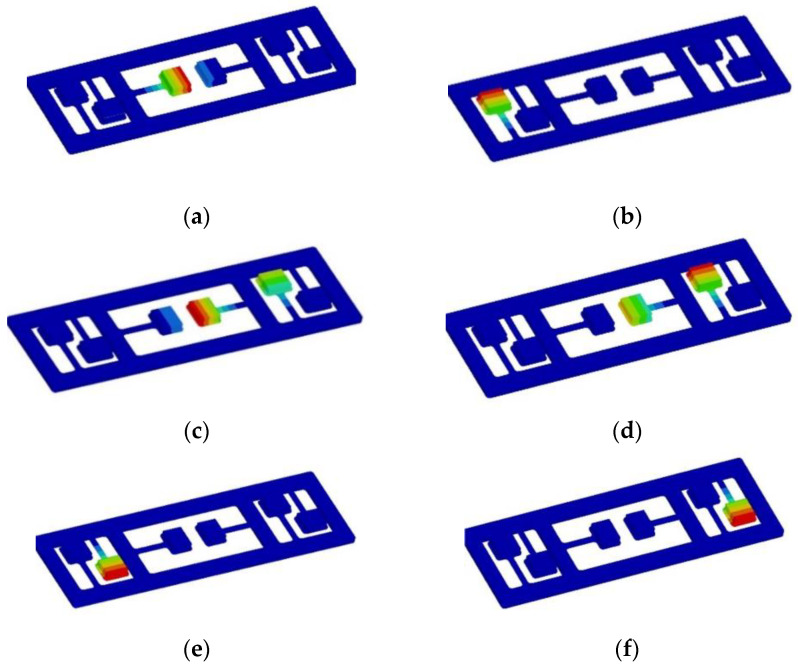
Natural frequency of acoustic metamaterial resonance element. (**a**) 34.94 Hz, (**b**) 34.94 Hz, (**c**) 34.95 Hz, (**d**) 34.95 Hz, (**e**) 35.00 Hz, (**f**) 35.00 Hz.

The mathematical model of acoustic metamaterial was imported into Hypermesh2017.01, which was processed by solid cutting and placed in the mapping state. A hexahedral mesh of 1mm was established for each cut entity. The total number of acoustic metamaterials grid is 40,324. Assign material attributes to each component, as shown in Table 3. After designing the modal solution card, it is imported into Nastran solver for solving the calculation. Based on the principle of local resonance of acoustic metamaterial, the resonance element has to produce local resonance with the sheet metal. The natural frequency of the resonant element is calculated as $f=\frac{1}{2\pi }\sqrt{\frac{k}{m}}$ (k is the equivalent stiffness, m is the equivalent mass). By adjusting the thickness of attached mass block 6 (which is equivalent to changing the value of f by changing m in the calculation formula of natural frequency), the first-order natural frequency of each resonance element in the acoustic metamaterial is within the range of 35 ± 0.5 Hz. The adjusted calculation results are shown in Figure 17. The natural frequencies of each resonance unit are, respectively, 34.94 Hz, 34.94 Hz, 34.95 Hz, 34.95 Hz, 35.00 Hz and 35.00 Hz.

## 4. Results and Discussion

**Figure 18:**
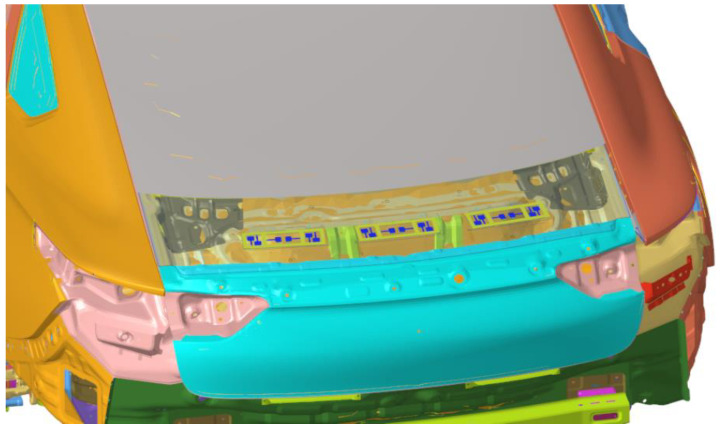
Acoustic metamaterial finite element model attached to the vehicle.

After the corresponding acoustic metamaterial is designed, the acoustic metamaterial finite element model is attached to the design part, and the whole vehicle finite element model with the acoustic metamaterial attached to the tailgate is established, as shown in Figure 18 (in order to see the installation position of the acoustic metamaterial, the attached sheet metal parts is displayed transparently). The response points are the sound pressure level in the driver’s right ear, the middle pressure level in the rear passenger’s seat and the vibration acceleration in the middle of the tailgate panel.

**Figure 19:**
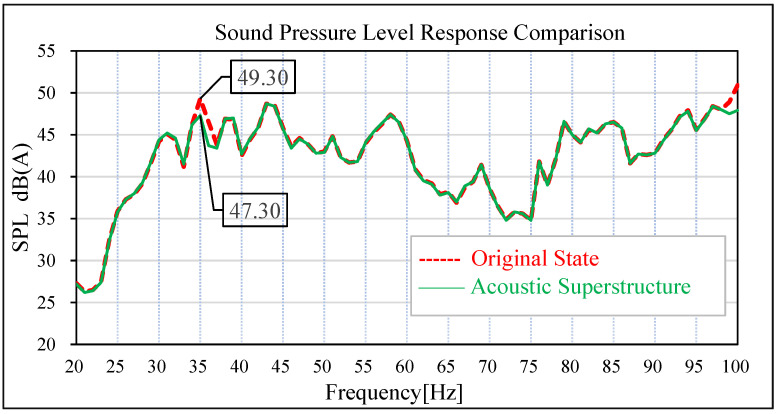
Comparison of sound pressure levels in the driver’s right ear.

**Figure 20:**
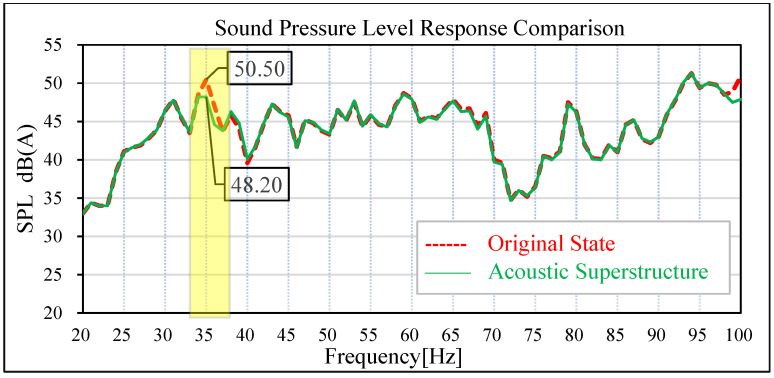
Comparison of sound pressure levels in the middle of rear passenger row.

**Figure 21:**
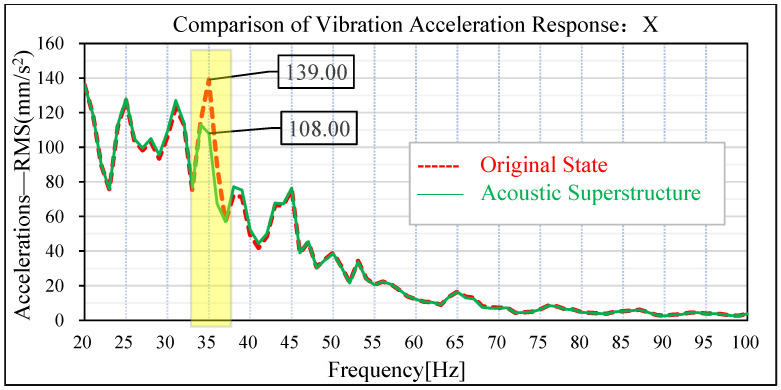
Comparison of x-direction vibration response of sheet metal parts of the tail door.

**Figure 22:**
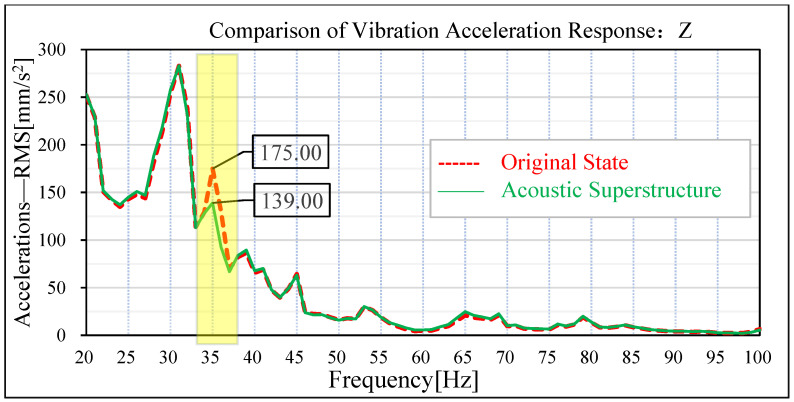
Comparison of z-direction vibration response of sheet metal parts of tail door.

The same road noise excitation signal and excitation point positions as in Section 3 were used to conduct road noise simulation analysis and vibration response analysis of the plate metal parts of the tailgate before and after the attaching of the acoustic–solid coupled finite element model. The simulation results are shown in Figure 19, Figure 20, Figure 21 and Figure 22. By comparing the data before and after the acoustic metamaterial, the vibration and noise reduction effect of the acoustic metamaterial was verified.

In the original state without an acoustic metamaterial attached, the sound pressure level at the driver’s right ear and the middle seat of the rear passenger’s seat has an obvious peak value at 35 Hz; the amplitude of the sound pressure level of the driver’s right ear at 35 Hz is 49.30 dB (A), and the amplitude of the sound pressure level of the passenger’s middle seat at 35 Hz is 50.50 dB (A). Under the condition of attached acoustic metamaterial, the amplitude of the sound pressure level in the driver’s right ear and the middle position of the passenger’s rear row is significantly reduced at 35 Hz. The amplitude of sound pressure level at 35 Hz in the driver’s right ear decreased from 49.30 dB (A) to 47.30 dB (A), and the improvement reached 2.0 dB (A). The amplitude of sound pressure level at 35 Hz in the middle of the passenger rear row decreased from 50.50 dB (A) to 48.20 dB (A), and the improvement reached 2.30 dB (A).

In the original condition without attached acoustic metamaterial, the vibration acceleration of the tailgate panel appears at an obvious peak value of 35 Hz. In the vehicle coordinate system, the amplitude of vibration acceleration of the tailgate panel at 35 Hz in the x-direction is 139 m/s^2^, and the amplitude of vibration acceleration at 35 Hz in the z-direction is 175 m/s^2^. Under the acoustic metamaterial condition, the amplitude of vibration acceleration of the tailgate panel at 35 Hz was significantly reduced. The amplitude of vibration acceleration at the x-direction of 35 Hz of the tailgate panel is reduced from 139 m/s^2^ to 108 m/s^2^, and the improvement reaches 31 m/s^2^. The amplitude of vibration acceleration at z-direction 35 Hz of the tailgate sheet metal parts decreased from 175 m/s^2^ to 139 m/s^2^, and the improvement reached 36 m/s^2^.

By comparing the data verified by the above simulation, it is found that the local resonant acoustic metamaterial has an ideal effect on the control of low-frequency noise radiated from the vibration of sheet metal parts to the vehicle.

Regarding the durability of the device, components of acoustic metamaterial: aluminum plate and the damping layer have good durability and have been widely used in the automotive industry, its durability can meet the needs of mass production applications. In addition to that, the three-interval method of random vibration fatigue life calculation based on Gaussian distribution and Miner’s linear cumulative damage criterion shows that the structure can carry out infinite cycles under the random load, which meets the engineering requirements.

## 5. Conclusions

Focusing on the problem of low-frequency noise in a certain car, this paper determines the frequency point of an abnormal peak value of noise in the car through test and simulation analysis. Through CAE simulation analysis, it is concluded that the low-frequency noise is caused by the low-frequency vibration of sheet metal parts of the tailgate. To solve this problem, an acoustic metamaterial is designed based on the band gap theory of local resonant phonon crystal. Through simulation verification, it is judged that the structure can indeed reduce the amplitude of interior noise and vibration acceleration, which verifies the effectiveness of the acoustic metamaterial. It provides a new way of thinking about the problem that the low-frequency noise of vibration and radiation of sheet metal parts is difficult to be controlled.

## Figures and Tables

**Table 1:** The relationship between system dynamic equivalent mass $m_{e}$ and excitation frequency *w*.

Excitation Frequency Range	Dynamic Equivalent Mass	System Movement Status
w=0	$m_{e}=M+m$	*M* and *m* move synchronously
$0<w<w_{0}$	$m_{e}>0$	*M* and *m* move in the same direction
$w\to w_{0}$	$m_{e}\to \infty$	*M* and m resonate, and the system vibration tends to be static
w0<w<w0(M+m)M	$m_{e}<0$	The equivalent mass is negative, *M* and *m* move in opposite directions
w→w0(M+m)M	$m_{e}\to 0$	Equivalent mass tends to 0, system resonance
w>w0(M+m)M	$m_{e}>0$	*M* and *m* move in the same direction
w→∞	$m_{e}\to M$	*m* tends to stand still

**Table 2:** Acoustic cavity modes table.

Order Time	Model Frequency	Model Vibration Shape
The first-order	49.98 Hz	First-order longitudinal
The second-order	94.63 Hz	First-order lateral

**Table 3:** Properties of acoustic metamaterial materials.

Part Name	Modulus of Elasticity (MPa)	Density (*T*/*mm*^3^)	Poisson’s Ratio
Acoustic metamaterial Matrix Plate 5	71,000	2.7 × 10^−9^	0.3
Adjustable Mass Block 6	210,000	7.9 × 10^−9^	0.3
Acoustic metamaterial Damping Layer 7	3457	2.1 × 10^−9^	0.49
